# Supplementary material for: RNA helicase DDX3X modulates herpes simplex virus 1 nuclear egress
Source: Commun Biol. 2023 Feb 1;6:134. doi: 10.1038/s42003-023-04522-w (PMC9892522; doi:10.1038/s42003-023-04522-w)
Supplement: Supplementary file 2 — Supplementary Information [file 42003_2023_4522_MOESM2_ESM.pdf]

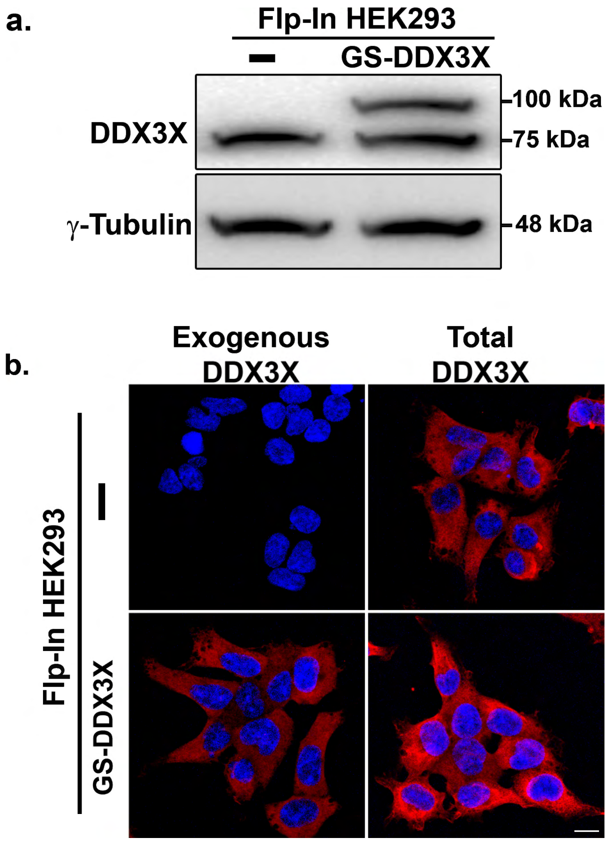


Fig S1: Analysis of tagged DDX3X stable cell line. HEK293 Flp-In cells stably expressing GS-DDX3X were analyzed by **a)** Western blot and **b)** immunofluorescence (conventional confocal fluorescence microscopy). **a)** HEK293 Flp-In cells with or without GS-DDX3X stable expression were lysed and the expression of DDX3X and GS-DDX3X was analyzed on an 8% SDS-PAGE using an anti-DDX3X antibody. γ-tubulin was used as the loading control. Note the similar expression of the exogenous (top band) and endogenous (lower band) DDX3X. **b)** HEK293 Flp-In cells with or without GS-DDX3X stable expression were grown on coverslips, fixed, permeabilized and labelled with Goat anti-Rabbit Alexa 568 to reveal the exogenous GS-tagged DDX3X (left panels) or with an anti-DDX3X antibody to detect the entire DDX3X pool (exogenous and endogenous; right panels). DDX3X is shown in red while nuclei were stained using Hoechst 33342 (blue). The exogenous DDX3X (bottom left panel) was similar to the endogenous DDX3X (top right panel). Scale bar represents 10 μm. The results represent three independent experiments.

**a.**

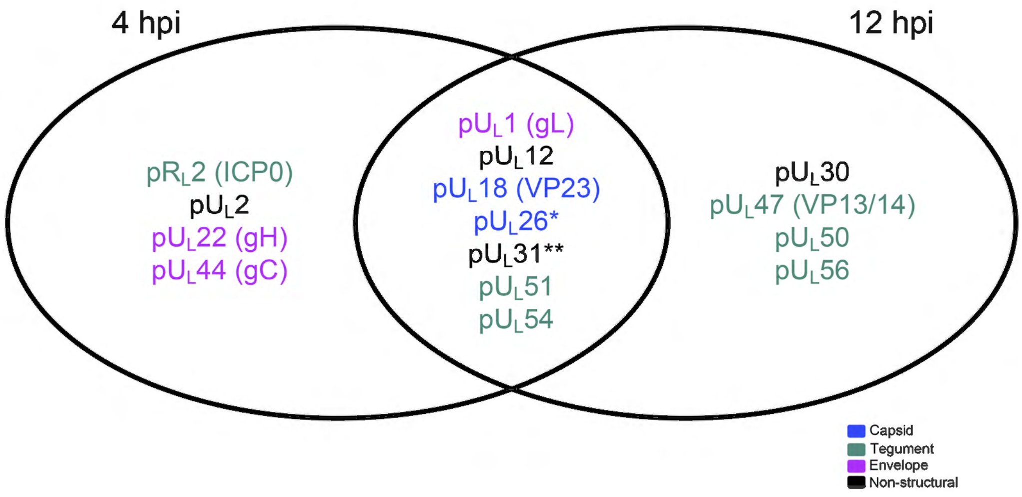


## b. Viral peptides identified

| | Gene name | Common protein name | MW | 4 hpi Exp 1 | 4 hpi Exp 2 | 4 hpi Exp 3 | 12 hpi Exp 1 | 12 hpi Exp 2 | 12 hpi Exp 3 | mock |
|---|---|---|---|---|---|---|---|---|---|---|
| **Capsid** | **$U_L18$** | VP23 | 34 kDa | 17 | 27 | 29 | 21 | 36 | 28 | none |
| | **$U_L26$** | $pU_L26$ | 66 kDa | 24 | 14 | 25 | 59 | 12 | 10 | none |
| **Tegument** | **$R_L2$** | ICPO | 78 kDa | 74 | 4 | 12 | 59 | 0 | 0 | none |
| | **$U_L47$** | VP13/14 | 74 kDa | 0 | 0 | 2 | 162 | 64 | 63 | none |
| | **$U_L50$*** ** | $pU_L50$ | 39 kDa | 64 | 12 | 21 | 76 | 15 | 19 | 2 (4 hpi) |
| | **$U_L51$** | $pU_L51$ | 25 kDa | 3 | 3 | 2 | 25 | 4 | 4 | none |
| | **$U_L54$** | $pU_L54$ | 55 kDa | 120 | 18 | 10 | 131 | 4 | 3 | none |
| | **$U_L56$** | $pU_L56$ | 25 kDa | 2 | 1 | 0 | 5 | 14 | 3 | none |
| **Envelope** | **$U_L1$** | gL | 25 kDa | 3 | 11 | 16 | 12 | 24 | 40 | none |
| | **$U_L22$** | gH | 90 kDa | 30 | 3 | 30 | 55 | 0 | 26 | none |
| | **$U_L44$** | gC | 54 kDa | 43 | 7 | 14 | 153 | 0 | 51 | none |
| **Non-structural** | **$U_L2$** | $pU_L2$ | 36 kDa | 12 | 2 | 15 | 10 | 0 | 1 | none |
| | **$U_L12$** | $pU_L12$ | 67 kDa | 35 | 7 | 15 | 35 | 7 | 17 | none |
| | **$U_L30$** | $pU_L30$ | 137 kDa | 9 | 0 | 4 | 15 | 4 | 11 | none |
| | **$U_L31$** | $pU_L31$ | 34 kDa | 10 | 4 | 5 | 47 | 28 | 27 | none |

Fig S2: DDX3X viral partners identified by mass spectrometry. **a)** HEK293 Flp-In cells stably expressing GS-DDX3X were grown in 150 mm dishes. The cells were mock-treated or infected with wild-type strain 17+ virus at an MOI of 5. At 4 or 12 hpi, cells were lysed and GS-DDX3X was enriched using IgG Sepharose beads. GS-DDX3X and its binding partners were released from the beads by boiling in sample buffer. The proteins were separated on a 5-15 % gradient SDS-PAGE, stained with Coomassie Blue and analyzed by MS/MS. Uninfected samples were used as controls. The results represent three independent experiments. * It should be noted that pUL26 encodes a viral protease that cleaves itself into VP21 and VP24 and that it is not possible to distinguish the full-length protein from the cleaved version by MS. ** pUL31 is technically a nonstructural viral protein since absent on mature extracellular virions. **b)** Total viral peptides identified by MS. ***For pUL50, two peptides were identified in the mock control at 4 hpi, so the protein was considered negative for that time.

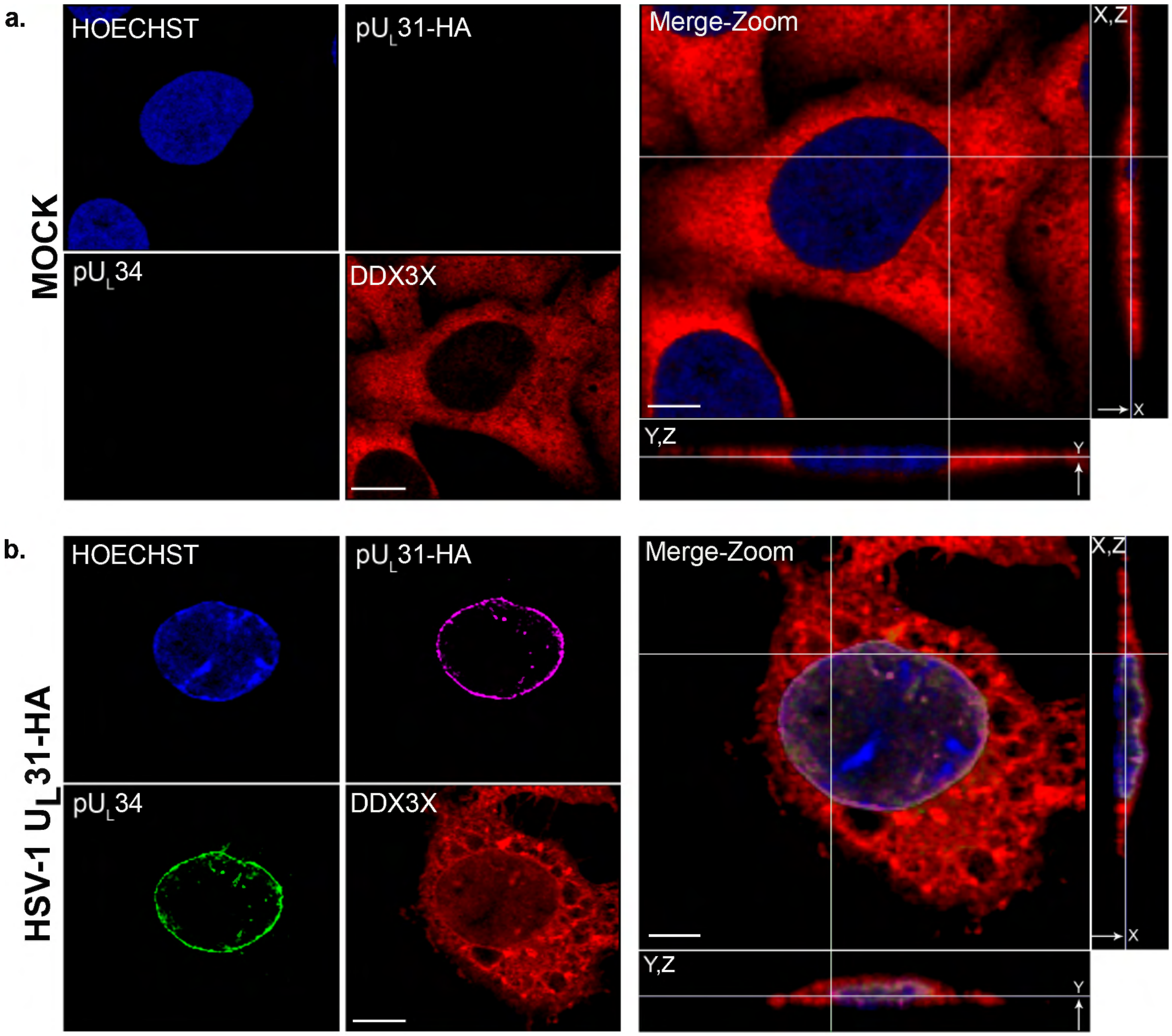


Fig S3: DDX3X co-localizes with pUL31 at the nuclear envelope. HeLa cells seeded on coverslips were **a)** mock-treated or **b)** infected with HA-tagged pUL31 virus for 9 hpi. Cells were immunolabelled with DDX3X (red), pUL31 (magenta) and pUL34 (green) antibodies while nuclei were stained using Hoechst 33342 (blue). The distribution of DDX3X around the nuclear rim in the absence and presence of infection was analyzed on orthogonal projections using Leica's LAS X software. Scale bare represents 5 μm. The images were acquired on a conventional confocal fluorescence microscopy. The results are representative of three independent experiments.

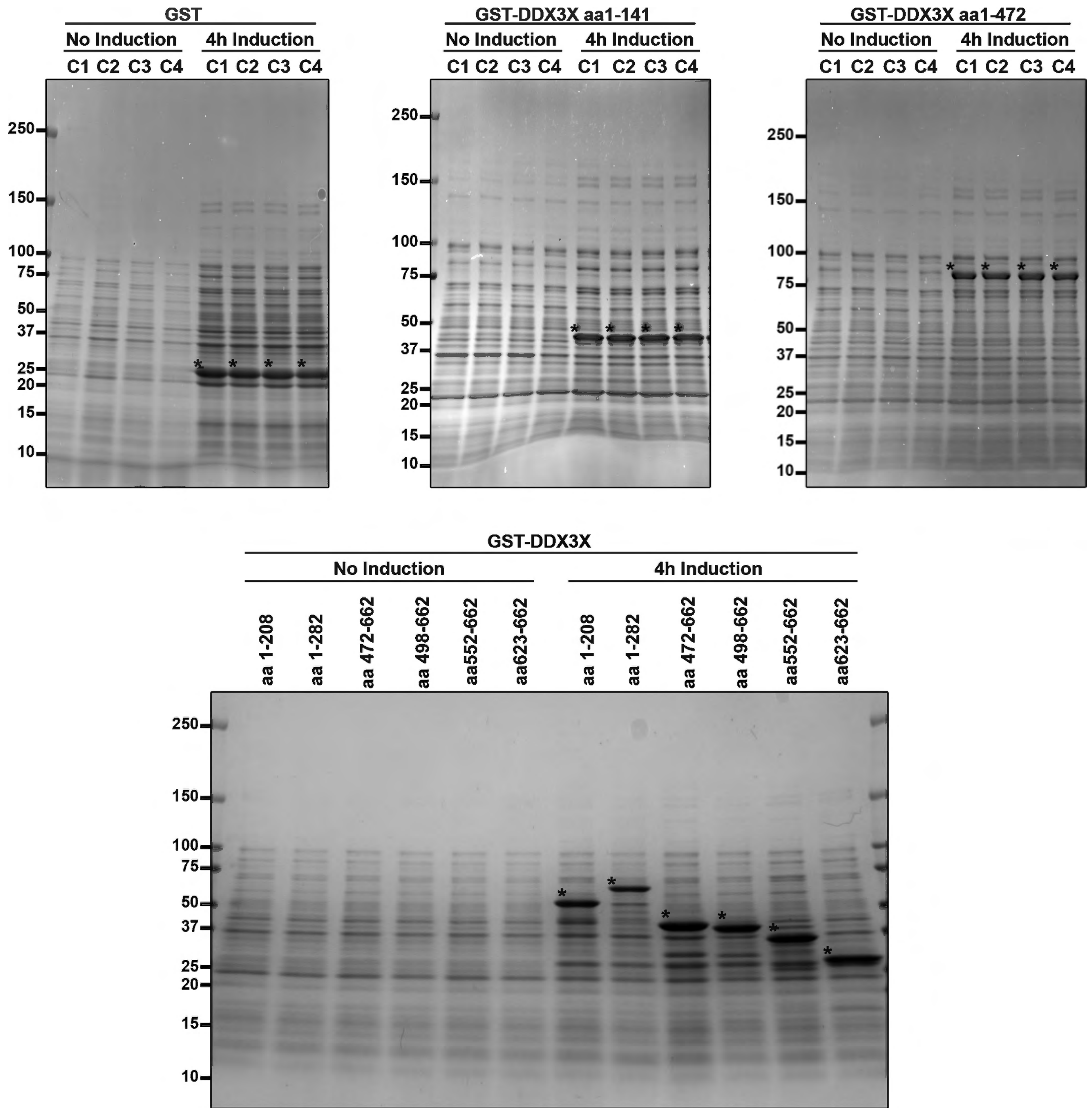


Fig S4: Expression levels of recombinant DDX3X fragments. BL21 bacteria transformed with an empty GST control vector or expressing different GST-tagged DDX3X recombinant constructs were grown in LB medium at 37°C until the OD600 reached 0.4 then IPTG induced for 4 h at 37°C. Crude bacterial aliquots prior and after induction were boiled in Laemmli sample buffer, analyzed by SDS-PAGE and Coomassie staining. Stars represent the position of each GST-tagged fragment and C1 to C4 the colony number. The molecular weights of the markers are indicated to the left of the gels.

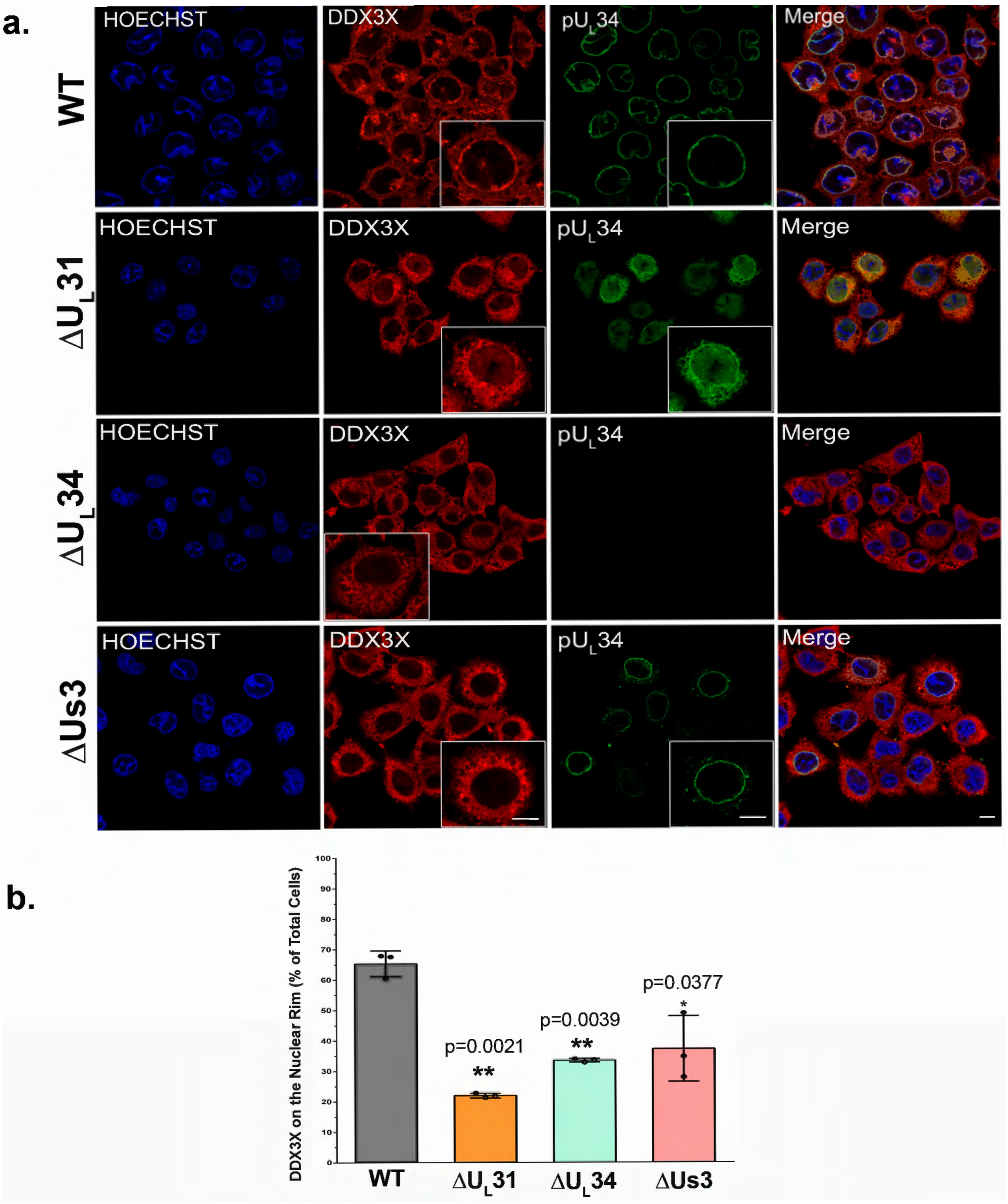


Fig S5: DDX3X nuclear membrane targeting depends on the viral nuclear egress machinery. **a)** HeLa cells that were grown on coverslips were either mock-treated or infected with the ΔUS3, ΔUL31 or ΔUL34 mutant viruses or corresponding wild-type virus (F strain) at an MOI of 5. Nine hours post-infection, the cells were processed for immunofluorescence (DDX3X: red; pUL34: green; nuclei stained with Hoechst 33342: blue) and imaged by conventional confocal microscopy. Zoom boxes were added to highlight DDX3X and pUL34 localization in each condition. **b)** Quantification of the above data using three independent experiments. The data depict the average number of cells that exhibited DDX3X nuclear rim staining. The error bar indicates the standard deviation of the means. All scale bars represent 10 μm. * $p<0.05$; ** $p<0.01$ (bilateral Student T tests).

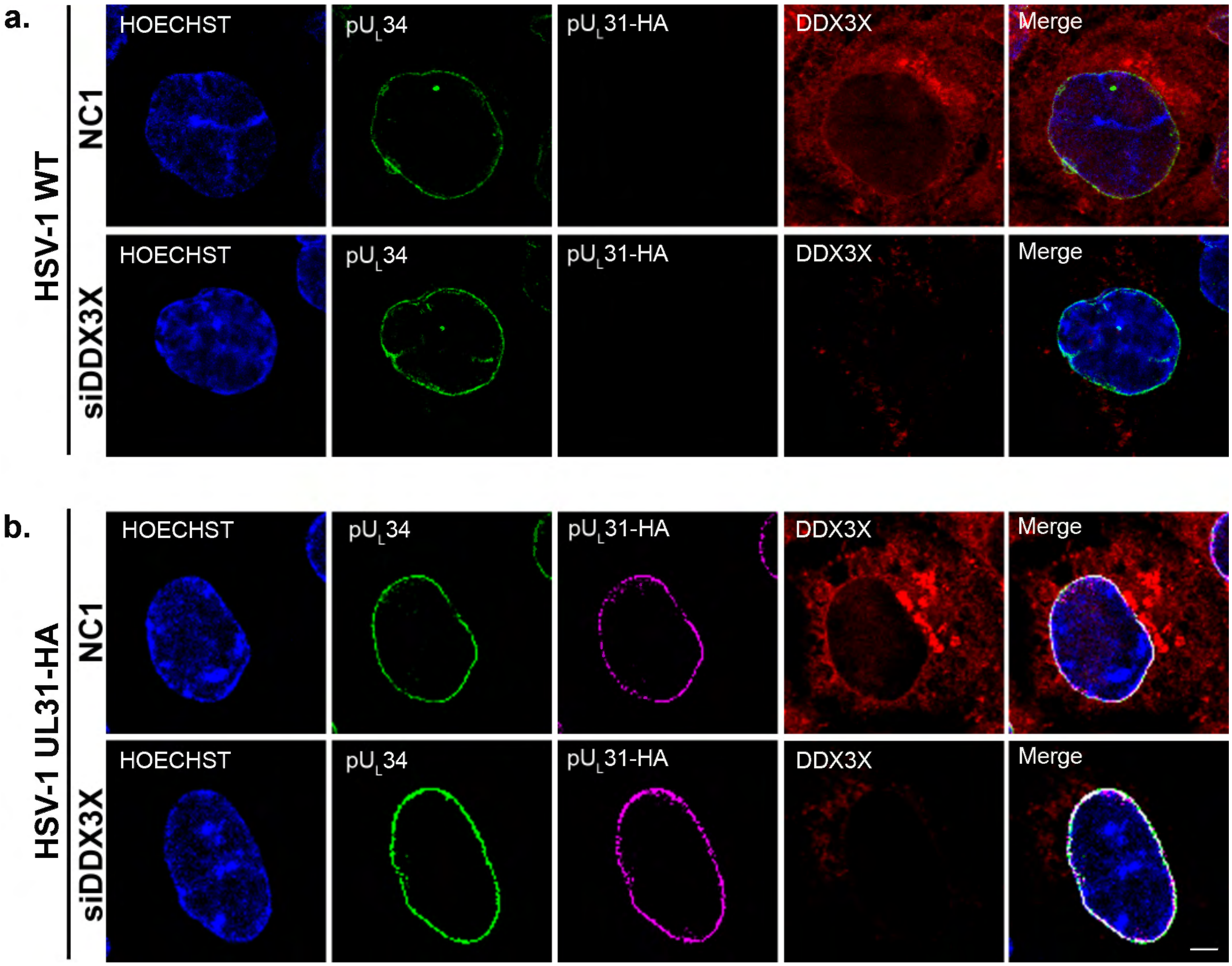


Fig S6: NEC nuclear localization is DDX3X independent. HeLa cells seeded on coverslips were treated with 25 nM of siDDX3X or NC1 siRNA for 48 h. The transfected cells were then infected with **a)** wild-type strain F virus or **b)** HA-tagged pUL31 virus at an MOI of 5 for 9 hpi. The fixed cells were labelled for DDX3X (red), pUL34 (green) and HA-tag (magenta). Nuclei were stained using Hoechst 33342 (blue). Scale bars represent 5 µm. The images were acquired by conventional confocal microscopy and are representative of three independent experiments. NC1: Not targeting siRNA control.

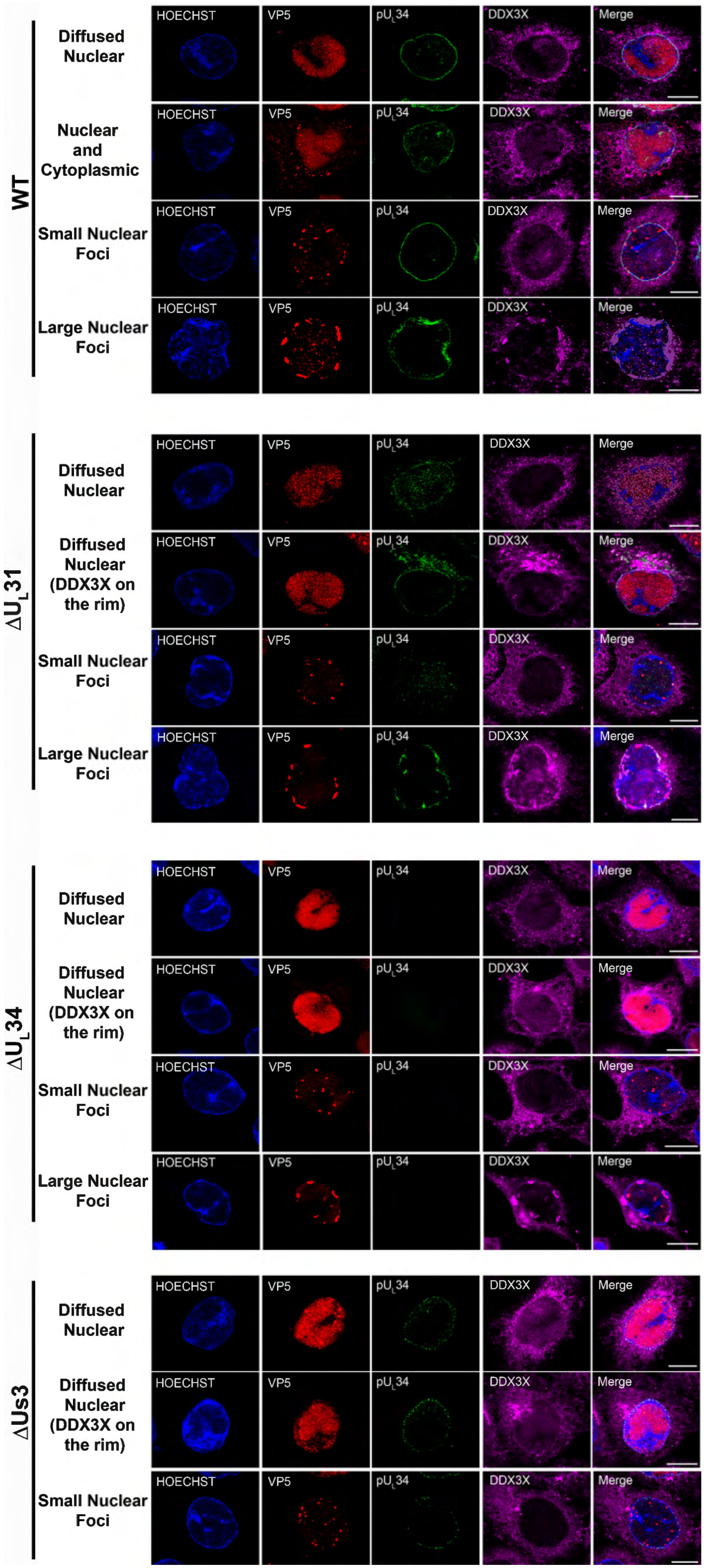


Fig S7: VP5 aggregates at the nuclear periphery are NEC dependent. HeLa cells were seeded on coverslips and then infected with the ΔUS3, ΔUL31 or ΔUL34 mutant viruses or corresponding wild-type virus (F strain) at an MOI of 5. At 9 hpi, cells were fixed and treated for conventional confocal microscopy to probe DDX3X (magenta), VP5 (red) and pUL34 (green). Images are representatives of three independent experiments. Scale bar represents 10 µm.

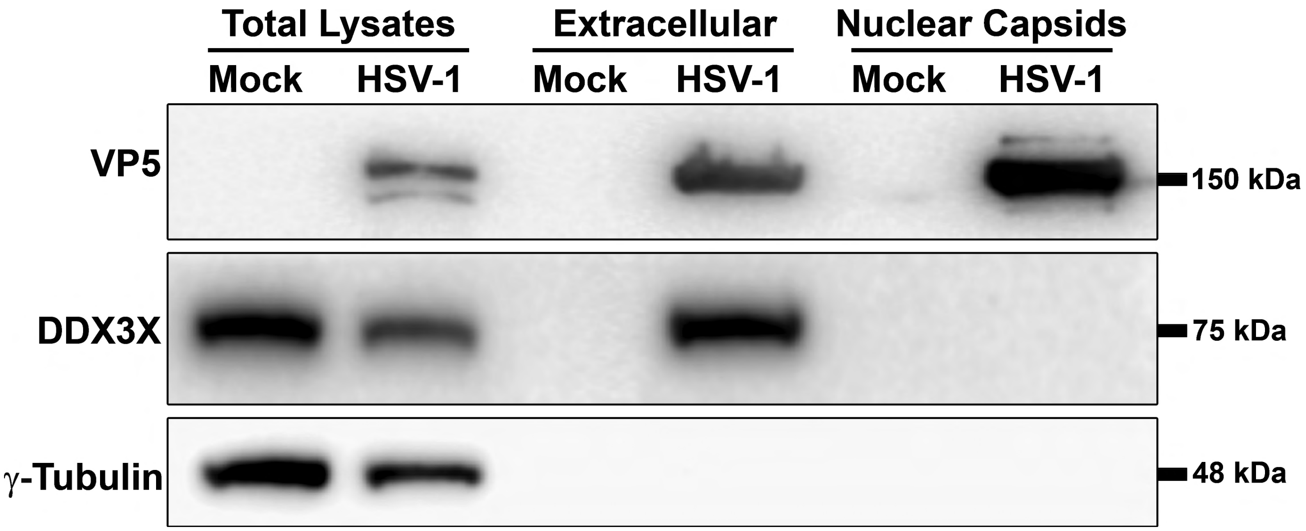


Fig S8: DDX3X is absent on nuclear capsids. HeLa cells were mock-treated or infected with wild-type strain 17+ virus at an MOI of 5. Twenty-four hours post-infection, the extracellular milieu was separated by low speed centrifugation. Total cell lysates were prepared by incubating the cell pellets with lysis buffer while extracellular virions were concentrated at 60,000 x g from the supernatant. Intact nuclei were isolated in parallel from other mock treated or infected cells, separated from the cytosol by centrifugation and subjected to freeze-thaw to release the nuclear capsids. The nuclear capsids were then enriched and concentrated on a 35% sucrose cushion. All samples were analyzed on 8% SDS-PAGE and Western blotting, probing DDX3X, the major viral capsid protein VP5 and, as loading control, γ-tubulin. The data represents three independent experiments.

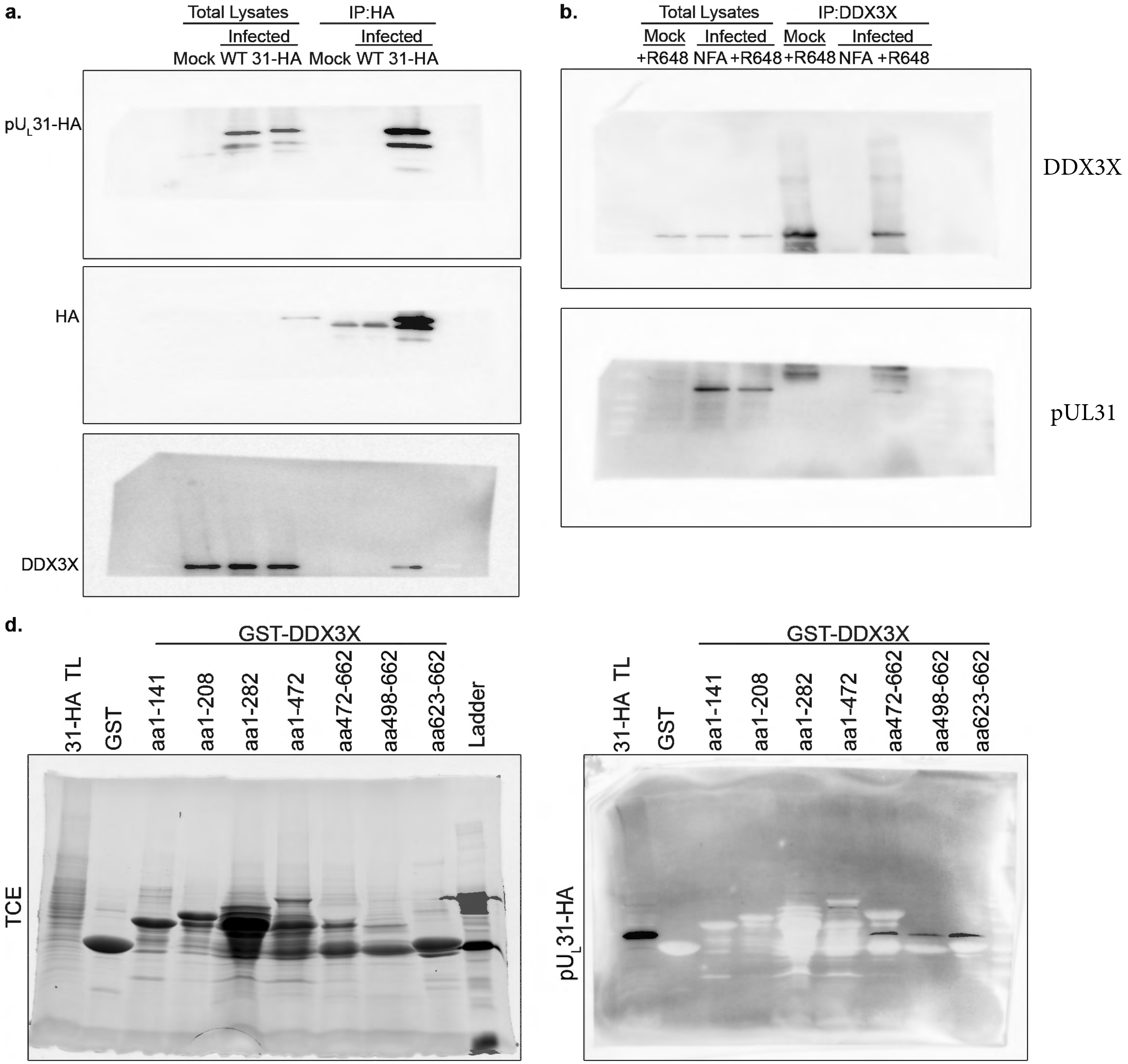


Fig S9: Original blots for figure 3a, b and d (panel d indicates two different exposures)

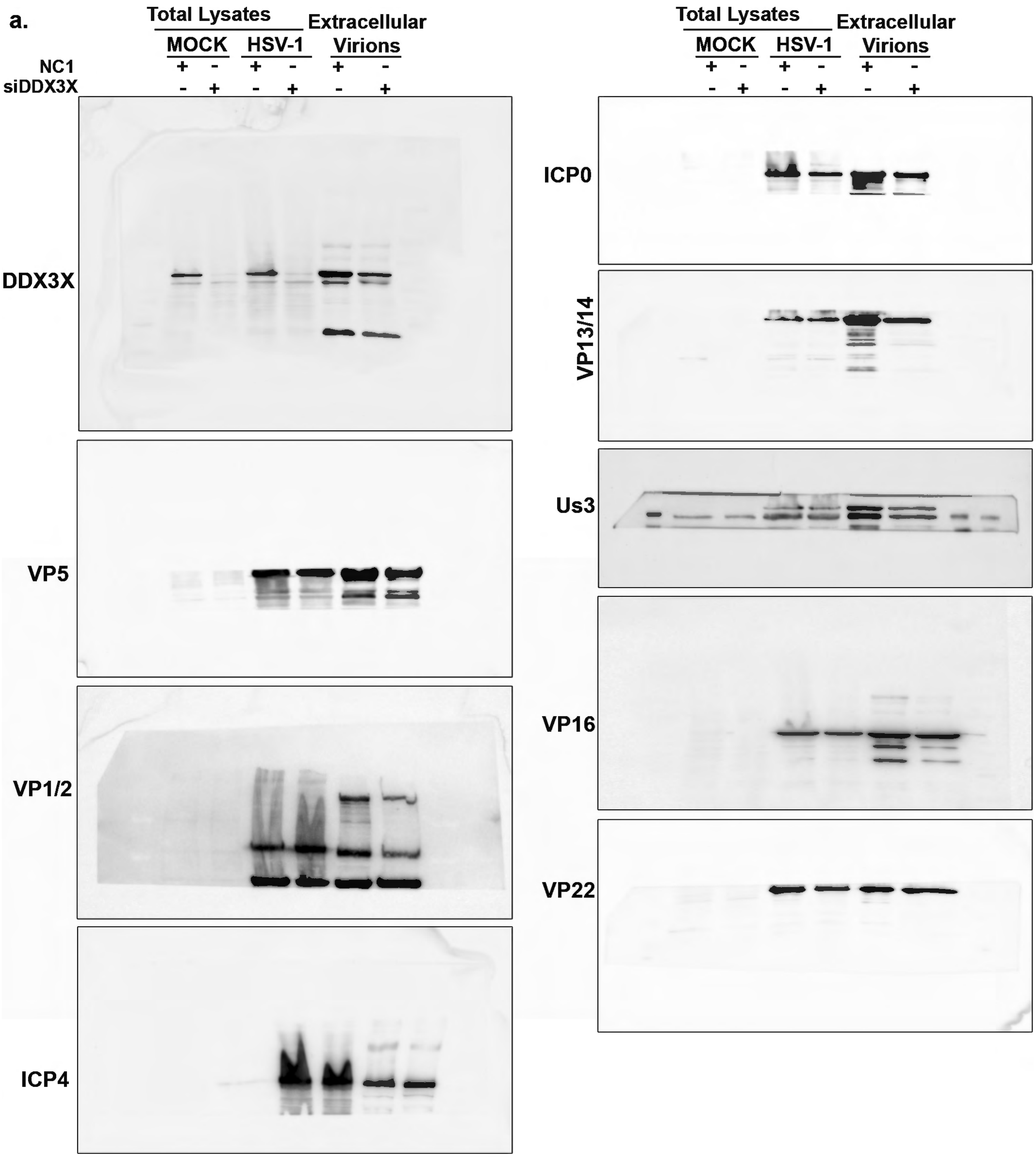


Fig S9: Original blots for figure 9a

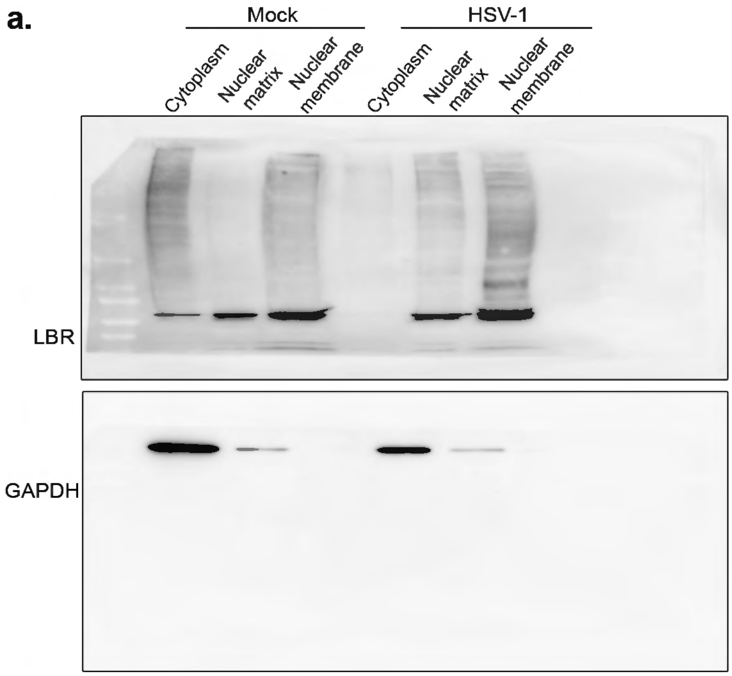


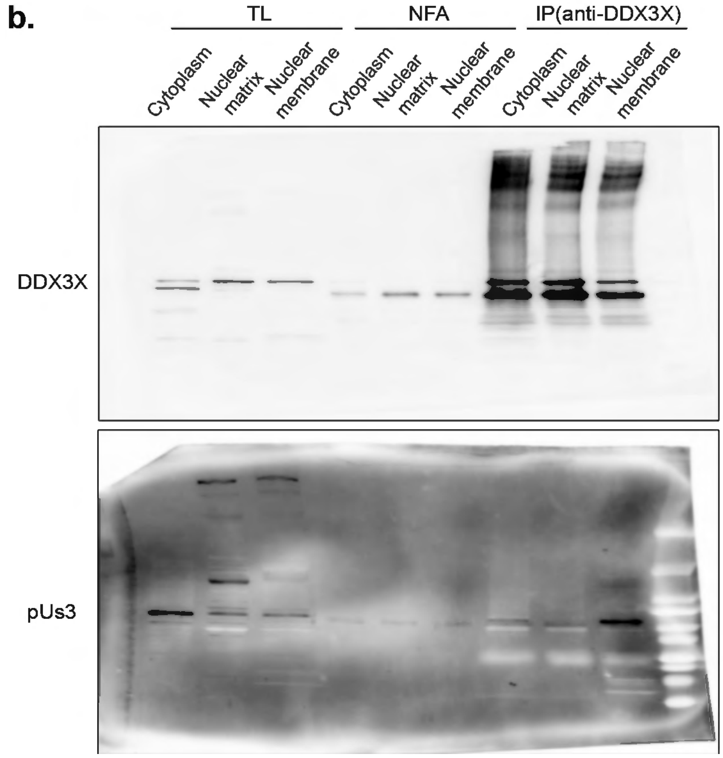


Fig S9: Original blots for figure 10a and b

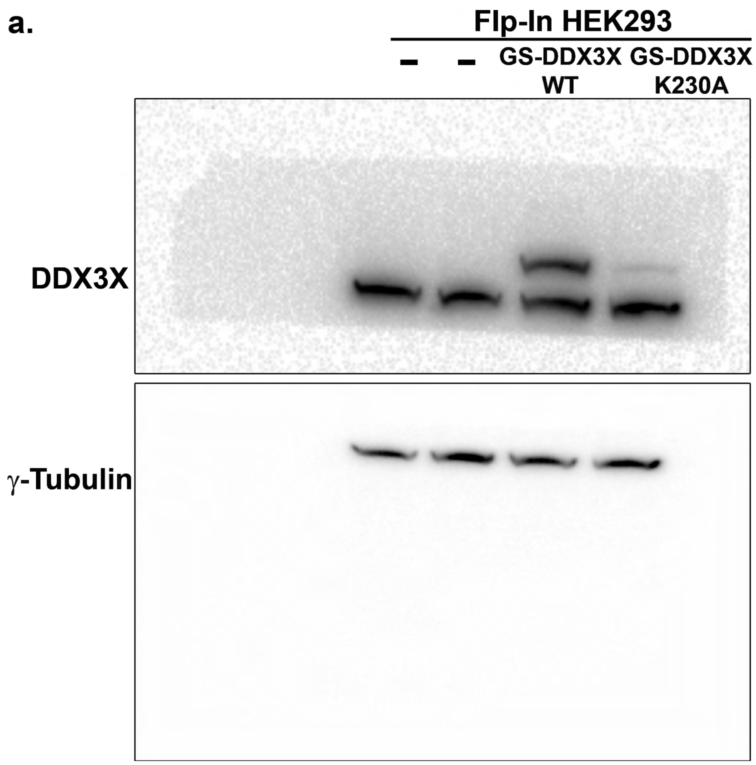


Fig S9: Original blots for figure S1a

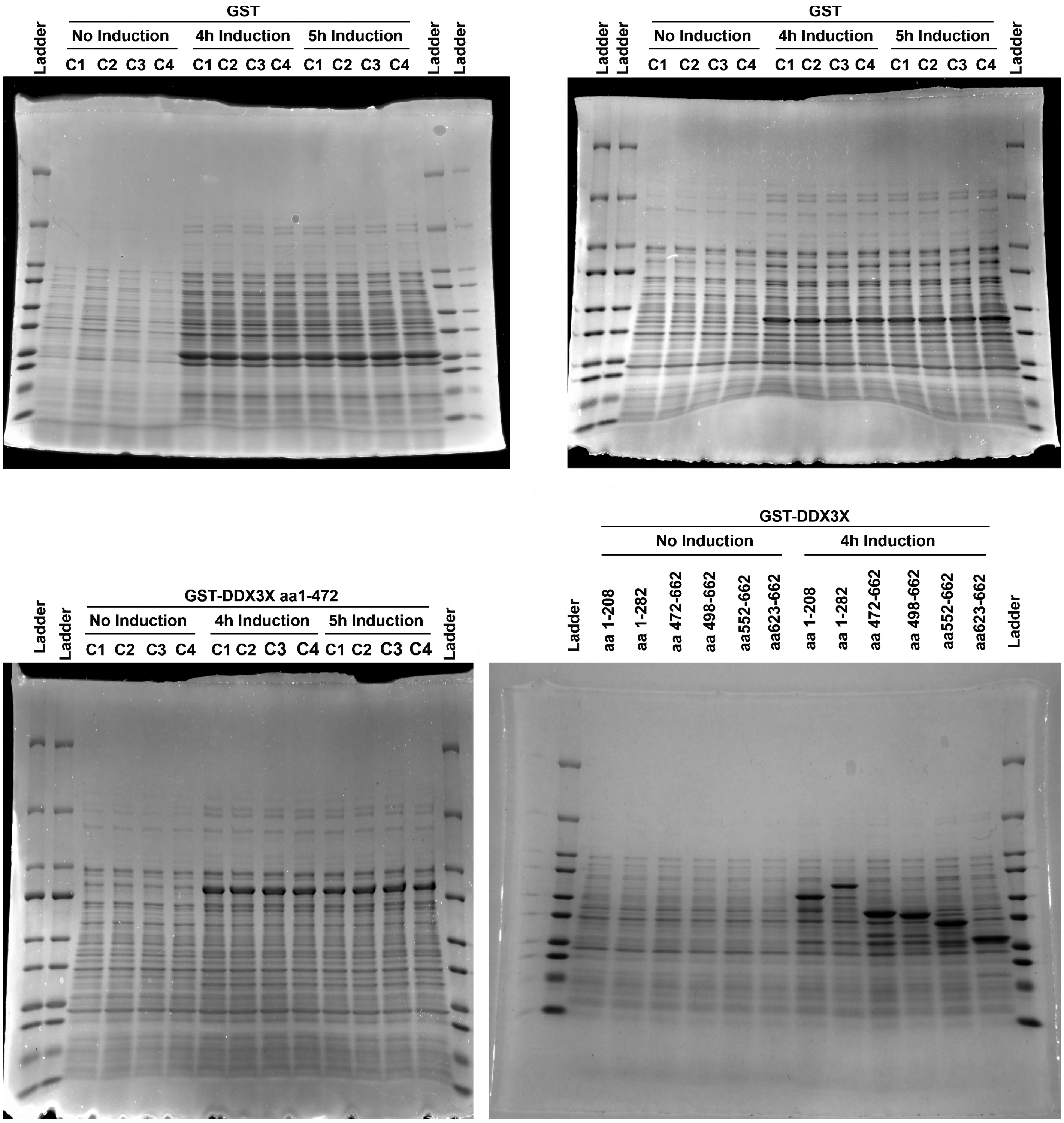


Fig S9: Original blots for figure S4

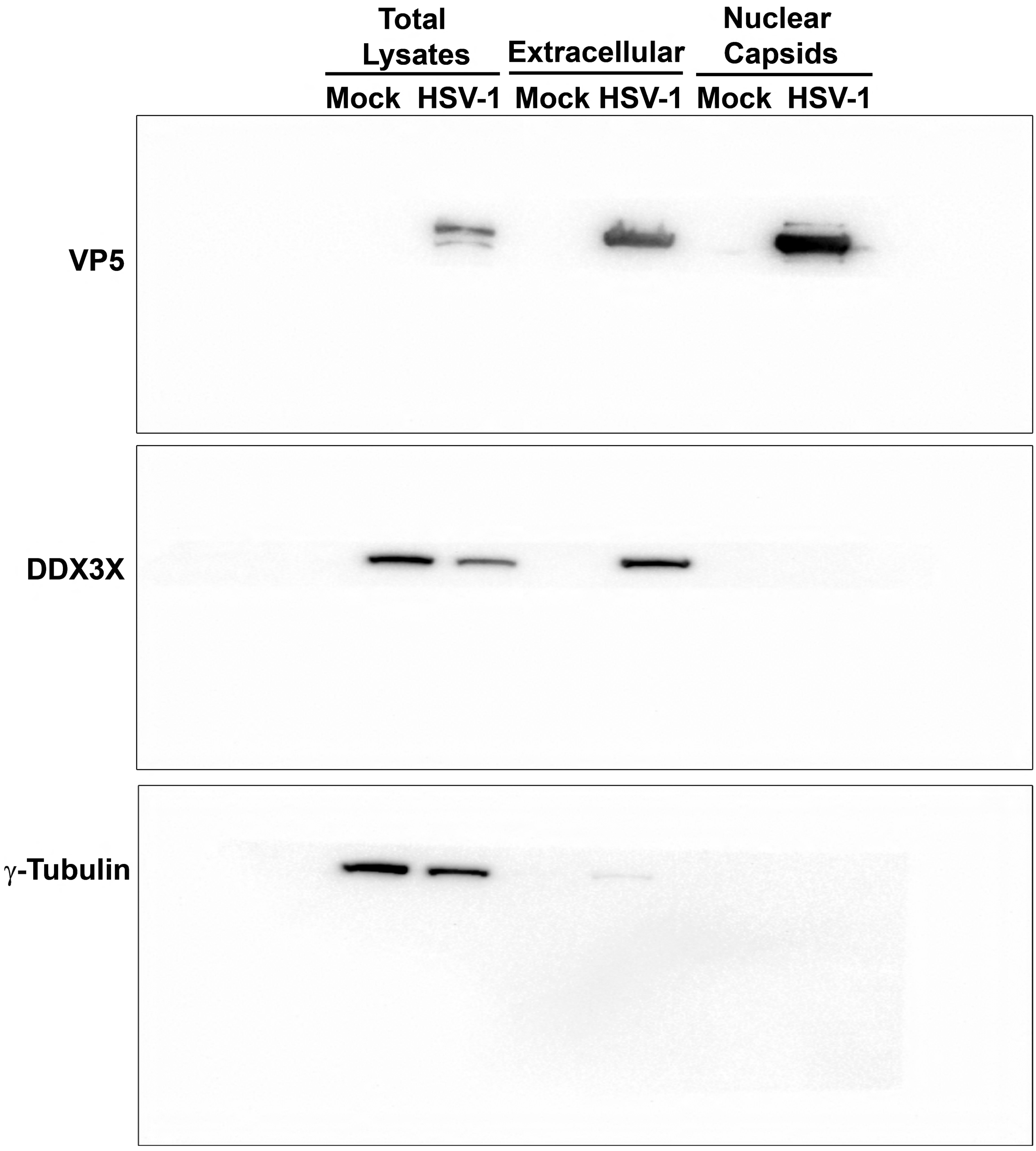


Fig S9: Original blots for figure S8
