# Supplementary material for: RNA helicase DDX3X modulates herpes simplex virus 1 nuclear egress
Source: Commun Biol. 2023 Feb 1;6:134. doi: 10.1038/s42003-023-04522-w (PMC9892522; doi:10.1038/s42003-023-04522-w)
Supplement: Supplementary file 5 — Reporting Summary [file 42003_2023_4522_MOESM5_ESM.pdf]

nature portfolio

Corresponding author(s): Roger Lippé

Last updated by author(s): Jan 16, 2023

# Reporting Summary

Nature Portfolio wishes to improve the reproducibility of the work that we publish. This form provides structure for consistency and transparency in reporting. For further information on Nature Portfolio policies, see our Editorial Policies and the Editorial Policy Checklist.

## Statistics

For all statistical analyses, confirm that the following items are present in the figure legend, table legend, main text, or Methods section.

| n/a | Confirmed | |
|---|---|---|
| ☐ | ☒ | The exact sample size (*n*) for each experimental group/condition, given as a discrete number and unit of measurement |
| ☐ | ☒ | A statement on whether measurements were taken from distinct samples or whether the same sample was measured repeatedly |
| ☐ | ☒ | The statistical test(s) used AND whether they are one- or two-sided<br>*Only common tests should be described solely by name; describe more complex techniques in the Methods section.* |
| ☒ | ☐ | A description of all covariates tested |
| ☒ | ☐ | A description of any assumptions or corrections, such as tests of normality and adjustment for multiple comparisons |
| ☐ | ☒ | A full description of the statistical parameters including central tendency (e.g. means) or other basic estimates (e.g. regression coefficient) AND variation (e.g. standard deviation) or associated estimates of uncertainty (e.g. confidence intervals) |
| ☒ | ☐ | For null hypothesis testing, the test statistic (e.g. *F*, *t*, *r*) with confidence intervals, effect sizes, degrees of freedom and *P* value noted<br>*Give P values as exact values whenever suitable.* |
| ☒ | ☐ | For Bayesian analysis, information on the choice of priors and Markov chain Monte Carlo settings |
| ☒ | ☐ | For hierarchical and complex designs, identification of the appropriate level for tests and full reporting of outcomes |
| ☒ | ☐ | Estimates of effect sizes (e.g. Cohen's *d*, Pearson's *r*), indicating how they were calculated |

*Our web collection on statistics for biologists contains articles on many of the points above.*

## Software and code

Policy information about availability of computer code

| | |
|---|---|
| Data collection | No software was used |
| Data analysis | PEAKS X (Bioinformatics solutions) was used to process the MS peptides<br>Scaffold 4.3.0 was used for subsequent MS analysis<br>Image Lab version 5.0 was used for Western blots<br>LAS X version 3.7.2.22383 was used to analyze fluorescence microscopy images<br>Image J version 1.53k was used to quantify some images<br>Imaris version 9.7.2 & 9.8.0 was used to analyze STED images |

For manuscripts utilizing custom algorithms or software that are central to the research but not yet described in published literature, software must be made available to editors and reviewers. We strongly encourage code deposition in a community repository (e.g. GitHub). See the Nature Portfolio guidelines for submitting code & software for further information.

## Data

Policy information about availability of data

All manuscripts must include a data availability statement. This statement should provide the following information, where applicable:

- Accession codes, unique identifiers, or web links for publicly available datasets
- A description of any restrictions on data availability
- For clinical datasets or third party data, please ensure that the statement adheres to our policy

The mass spectrometry proteomics data have been deposited to the ProteomeXchange Consortium via the PRIDE [1] partner repository with the dataset identifier PXD039403 and 10.6019/PXD039403

## Human research participants

Policy information about studies involving human research participants and Sex and Gender in Research.

| | |
|---|---|
| Reporting on sex and gender | The present study was entirely done in tissue culture so gender is not an issue. However, HeLa (female cell line) were used, so sex may be an important variable. DDX3X is also an X-linked gene and is therefore present in two copies rather than one in male cells. |
| Population characteristics | No human participants or tissues were used in this study. |
| Recruitment | n/a |
| Ethics oversight | n/a |

Note that full information on the approval of the study protocol must also be provided in the manuscript.

# Field-specific reporting

Please select the one below that is the best fit for your research. If you are not sure, read the appropriate sections before making your selection.

☒ Life sciences ☐ Behavioural & social sciences ☐ Ecological, evolutionary & environmental sciences

For a reference copy of the document with all sections, see nature.com/documents/nr-reporting-summary-flat.pdf

# Life sciences study design

All studies must disclose on these points even when the disclosure is negative.

| | |
|---|---|
| Sample size | The number of independent experiments (n) is indicated in each figure legend |
| Data exclusions | No data were excluded from this study |
| Replication | Experiments were independently repeated three times |
| Randomization | n/a |
| Blinding | n/a |

# Reporting for specific materials, systems and methods

We require information from authors about some types of materials, experimental systems and methods used in many studies. Here, indicate whether each material, system or method listed is relevant to your study. If you are not sure if a list item applies to your research, read the appropriate section before selecting a response.

## Materials & experimental systems

| n/a | Involved in the study |
|---|---|
| ☐ | ☒ Antibodies |
| ☐ | ☒ Eukaryotic cell lines |
| ☒ | ☐ Palaeontology and archaeology |
| ☒ | ☐ Animals and other organisms |
| ☒ | ☐ Clinical data |
| ☒ | ☐ Dual use research of concern |

## Methods

| n/a | Involved in the study |
|---|---|
| ☒ | ☐ ChIP-seq |
| ☒ | ☐ Flow cytometry |
| ☒ | ☐ MRI-based neuroimaging |

# Antibodies

Antibodies used

The following non-commercial antibodies were used: anti-human DDX3X rabbit R648 polyclonal serum (from Arvind Patel); anti-HSV-1 pUL34 chicken polyclonal serum (from Richard Roller); anti-HSV-1 VP22 chicken polyclona & anti-HSV-1 pUL31 rabbit polyclonal (from Joel Baines); anti-HSV-1 VP13/14 rabbit polyclonal serum (from Gill Elliott); anti-HSV-1 VP16 mouse monoclonal antibody (from Helena Browne); anti-HSV-1 pUs3 rabbit polyclonal serum (from Bernard Roizman); rabbit polyclonal serum against HSV-1 capsids (from Beate Sodeik and Ari Helenius); anti-HSV-2 pUS3 rat polyclonal cross reacting against its HSV-1 homolog (from Bruce Banfield). The following primary commercial antibodies were also used: Anti-VP5 mouse monoclonal (EastCoast Bio Cat#HA018), LBR mouse monoclonal antibody (Abcam Cat#ab232731), GAPDH mouse monoclonal antibody (Millipore Sigma Cat#MAB374), γ-Tubulin mouse monoclonal antibody (Millipore Sigma Cat#T6557), HA-tag mouse monoclonal antibody (Santa Cruz Cat#sc-7392), mouse monoclonal ICP4 (Abcam Cat#ab6514) and ICP0 (Abcam Cat#ab6513). HRPO-Conjugated secondary antibodies were purchased from Bethyl Laboratories (Goat anti-Rabbit Cat#A120-201P), Jackson ImmunoResearch (Goat anti-Mouse Cat#115-035-003) or Cedarlane (Donkey anti-Chicken Cat#703-035-155). For immunofluorescence, Goat anti-Chicken Alexa Fluor 488 (Cat#A-11039), Goat anti-Rabbit Alexa Fluor 568 (Cat#A-11036), Chicken anti-Mouse Alexa Fluor 488 (Cat#A-21200) and Donkey anti-Mouse Alexa Fluor 647 (Cat#A-31571) were purchased from Invitrogen. For STED microscopy, Goat-Anti-Rabbit-IgG-Atto 647N (Sigma-Aldrich Cat#40839) and Goat anti-Mouse IgG (H+L) cross-adsorbed secondary antibody, Alexa Fluor 594 (Thermo Fisher Scientific Cat#A-11005) were used.

Validation

We rely in part on the validation by the manufacturer and confirmed them using, for example mock vs infected cell lysates for viral antibodies. Many of the antibodies are from labs (see Antibodies subsection in Methods) that already validated them in the litterature.

# Eukaryotic cell lines

Policy information about cell lines and Sex and Gender in Research

Cell line source(s)

HeLa

Authentication

The HeLa cell line was not authenticated

Mycoplasma contamination

We test all incoming cell lines for mycoplasma. In addition, we routinely check that our cell line remain mycoplasma free (commercial PCR kit covering numerous mycoplasma species)

Commonly misidentified lines
(See ICLAC register)

n/a
